# Non-obstructive cardiovascular disease: a new challenge for invasive cardiology?

**DOI:** 10.1007/s12471-017-1062-4

**Published:** 2017-11-29

**Authors:** M. E. Wittekoek, J. J. Piek

**Affiliations:** 1 HeartLife klinieken, Utrecht, The Netherlands; 2 AMC Heart Center, Academic Medical Center, Amsterdam, The Netherlands

Cardiovascular disease is the leading cause of death worldwide, and coronary artery disease (CAD) is the most common type of heart disease. Up to 20–30% of patients presenting with chest discomfort demonstrate no signs of obstructive CAD. In the field of invasive cardiology more than 50% of the patients undergoing coronary angiography for non-acute chest pain have normal or near normal coronary arteries [1]. Non-obstructive CAD in acute coronary syndromes has attracted much attention because of the relatively high incidence of adverse cardiovascular events if not appropriately diagnosed and treated [2].

Especially women have severe symptoms in the absence of obstructive coronary artery disease. Unfortunately, these women are often undertreated due to a lack of correct diagnosis and are at a three times higher risk (as compared with men) to experience a cardiac event within the first year after cardiac catheterisation [3]. In general, patients suffering from chest pain without obstructive coronary artery disease are often diagnosed as non-cardiac, such as gastrointestinal or psychiatric disorders.

It is important to diagnose angina in non-obstructive CAD due to concomitant epicardial spasm or microvascular dysfunction. Intracoronary acetylcholine provocation test (ACh test) is the preferred diagnostic method to document epicardial or microvascular function. However, this diagnostic procedure is rarely performed in clinical routine in the USA and Europe, because of the anticipated cardiac risk. Epicardial and microvascular spasm are considered to be due to endothelial dysfunction in the early phase of atherosclerosis characterised by intima hyperplasia. This results in inadequate nitrate oxide production by the endothelium on provocative stimuli causing smooth muscle cell contraction of the underlying media [4]. The treatment of coronary artery spasms consists of the administration of vasodilators, such as calcium channel blockers and nitrates, while the use of beta blockers is contra-indicated.

The presence of microvascular disease, which is more prevalent in women, has not comprehensively been studied in clinical practice. In a study of 32,856 patients presenting for their first cardiac catheterisation with suspected ischaemic heart disease, 23.3% of women versus 7.1% of men were found to have normal coronary arteries at angiography [5]. Another study found that among 886 patients who were referred for chest pain and subsequently underwent angiography, non-obstructive coronary arteries was five times more frequently present in women than in men (41% versus 8%) [6]. It is well documented that women in the peri- or postmenopausal period more frequently suffer from anginal complaints in the absence of obstructive CAD [7]. In another study of 99 patients with angina and non-obstructive coronary arteries, the mean age of women at diagnosis was 48.5 years and 61.5% of women were postmenopausal [8].

Classical risk factors such as smoking, hypertension and dyslipidaemia play an important role in CAD. Individuals with microvascular disease have a higher likelihood of presenting with features of the metabolic syndrome (e. g. hypertension, dyslipidaemia, and insulin resistance) than the general population (30% versus 8%, respectively). In addition, these patients show more frequently endothelium-dependent and -independent cutaneous microvascular dysfunction [9]. Risk factors such as pre-eclampsia, migraine, early menopause, polycystic ovarium syndrome and rheumatic diseases all predispose to disturbed epicardial and microvascular coronary dysfunction.

Evolving knowledge regarding sex differences in coronary heart disease is emerging. Prevalence, symptom manifestation, and pathophysiology of CAD vary between women and men. Cardiovascular mortality is the leading cause of death in women and therefore a better insight into the pathophysiology of CAD is required.

The present study on coronary vasospasm published in this journal is an important contribution to further understand the long-term outcome of this coronary syndrome in non-obstructive CAD [10]. Most of the information on this topic stems from Asian patient populations, while the information in Caucasians is rather scarce, in particular in those presenting with an acute coronary syndrome. Even though the number of patients studied is rather small, it demonstrates the benign clinical outcome under optimal medical therapy and surveillance. In addition, it illustrates the usefulness of acetylcholine provocation testing in this cohort of patients to confirm the diagnosis [https://youtu.be/Jm13PEP20eg].

The number of women presenting to our emergency wards with chest pain not due to obstructive CAD is growing. In a recent study of Ong et al., a total of 921 consecutive patients were evaluated to undergo a diagnostic angiogram that showed non-obstructive CAD. A positive acetylcholine provocation test was noted in 33% of the patients, illustrating the frequent finding of epicardial spasm or microvascular dysfunction as the underlying cause of the clinical presentation. The occurrence of epicardial spasm is similar in men and women, while diffuse or microvascular spasm is four times more frequent in women than in men. Moreover, this study showed that acetylcholine testing is safe to assess coronary vasomotor function [11]. With a clear and reassured diagnosis of coronary vasospasm, appropriate medical therapy can be installed to reduce morbidity and mortality. The present study in this journal, as well as the study of Ong et al., should encourage interventionalists to add the acetylcholine provocation test to their diagnostic armamentarium.
